# Adjuvant Radiotherapy for Groin Node Metastases Following Surgery for Vulvar Cancer: A Systematic Review

**DOI:** 10.3389/or.2024.1389035

**Published:** 2024-05-07

**Authors:** Federico Ferrari, Lamiese Ismail, Ahmad Sabbagh, Kieran Hardern, Robert Owens, Elisa Gozzini, Hooman Soleymani Majd

**Affiliations:** ^1^ Department of Clinical and Experimental Sciences, University of Brescia, Brescia, Italy; ^2^ Department of Obstetrics and Gynaecology, Oxford University Hospitals NHS Trust, Oxford, United Kingdom; ^3^ Department of Clinical Oncology, Oxford University Hospitals NHS Trust, Oxford, United Kingdom; ^4^ Department of Anaesthetics, University Hospital Bristol and Western Hospital, Bristol, United Kingdom; ^5^ Department of Gynaecology Oncology, Oxford University Hospitals NHS Trust, Oxford, United Kingdom

**Keywords:** vulvar cancer, radiotherapy, inguinofemoral lymph nodes, pelvic lymphadenectomy, morbidity

## Abstract

**Background:** Lymph node metastasis in vulvar cancer is a critical prognostic factor associated with higher recurrence and decreased survival. A survival benefit is reported with adjuvant radiotherapy but with potential significant morbidity. We aim to clarify whether there is high-quality evidence to support the use of adjuvant radiotherapy in this setting.

**Objectives:** The aim of the study was to assess the effectiveness and safety of adjuvant radiotherapy to locoregional metastatic nodal areas.

**Search Methods:** We conducted a comprehensive and systematic literature search of MEDLINE, Embase, Cochrane Central Register of Controlled Trials, Google Scholar, ClinicalTrials.gov, and the National Cancer Institute. We considered only randomized controlled trials (RCTs).

**Main Results:** We identified 1,760 records and finally retrieved only one eligible RCT (114 participants with positive inguinofemoral lymph nodes). All women had undergone radical vulvectomy and bilateral inguinal lymphadenectomy and had been randomized to adjuvant radiotherapy or to intraoperative ipsilateral pelvic lymphadenectomy without adjuvant radiotherapy. At 6 years, the overall survival (OS) was 51% versus 41% in favor of radiotherapy (HR 0.61; 95% CI 0.30–1.3) without significance and with very low certainty of evidence. At 6 year, the cumulative incidence of cancer-related deaths was 29% versus 51% in favor of adjuvant radiotherapy (HR 0.49; 95% CI 0.28–0.87). Recurrence-free survival at 6 years was 59% after adjuvant radiotherapy versus 48% after pelvic lymphadenectomy (HR 0.39; 95% CI 0.17–0.88). Three (5.3%) versus 13 (24.1%) groin recurrences were noted, respectively, in the adjuvant radiotherapy and pelvic lymphadenectomy groups. There was no significant difference in acute toxicities for pelvic lymphadenectomy compared to radiotherapy. In women with positive pelvic lymph nodes (20%), the OS at 6 year was 36% compared with 13% in favor of adjuvant radiotherapy. Late cutaneous toxicity rate appeared to be greater after radiotherapy (19% vs. 15%) but with less chronic lymphedema (16% vs. 22%).

**Conclusion:** There is only very low-quality evidence on administering adjuvant radiotherapy for inguinal lymph node metastases. Although the identified study was a multicenter RCT, there was a reasonable imprecision and inconsistency because of small study numbers, wide confidence intervals in the data, and early trial closure, resulting in downgrading of the evidence.

## Introduction

Cancer of the vulva is a rare disease with an annual incidence of 2–3 per 100,000 women, making up 5% of all female genital tract cancers [1]. Vulvar cancer incidence is strongly related to age and reaches its highest incidence rate in groups where patients are aged over 90 years [2]; 75%–90% of these incidences are found to be squamous cell carcinoma, with the majority of patients diagnosed at the early stage of disease [3]. Overall, 30% of women presenting with vulvar cancer will have nodal metastases [4]. Nodal involvement and surgical margin status are the two most important prognostic factors for local and distant recurrence, representing the two main factors analyzed for recommending adjuvant therapy [5]. Moreover, in women with resectable disease without nodal involvement, the 5-year OS rate is more than 80%, while it falls dramatically to less than 40% in women with inguinal nodal involvement, and if the cancer has spread to more distant lymph nodes in the pelvis (iliac or other pelvic lymph nodes), then the 5-year survival drops to as low as 10%–15% [1].

The most recent guidelines of the European Society of Gynecological Oncology (ESGO) and National Comprehensive Cancer Network (NCCN) recommend that the primary tumor should be removed by radical local excision to get a unique orientable piece with sufficient tumor-free margin [6]. Tumors with depth invasion ≤1 mm, according to the eighth version of the TNM classification [7], do not require groin treatment, while for unifocal tumors <4 cm without suspicious inguinofemoral lymph nodes on clinical examination and imaging, the SLN procedure is recommended. For tumors ≥4 cm and/or in the case of multifocal invasive disease, inguinofemoral lymphadenectomy (IFL) by separate incisions is mandatory except in lateralized tumors in which ipsilateral IFL should be performed [8, 9].

Adjuvant radiotherapy treatment is administered with the main goal to reduce the incidence of local and inguinofemoral and pelvic recurrence that are often fatal [10–12]. Radiotherapy to the vulva after surgery is advised for all women who have a positive margin and cannot undergo further surgery to remove it. Additionally, radiotherapy might be an option when there are tumor characteristics that could increase the risk of recurrence, even if its role is controversial [13, 14]. The most important risk factors identified are narrow margins, in particular margins closer than 3 mm [15]; large size of tumors [16]; poorly differentiated tumors [17]; and/or tumors that have penetrated more than 5 mm deep [18–20]. Additionally, the GROINSS-V-II study proved that patients with SLN metastasis ≤2 mm can be treated with post-operative radiotherapy omitting ipsilateral inguinofemoral dissection with a 2-year isolated groin recurrence rate less than 1.6%, while patients with early stage vulvar cancer with SLN macrometastasis should undergo IFL followed by post-operative radiotherapy in case of one or more additional lymph node metastasis and/or extracapsular tumor spread; the 2-year isolated groin recurrence rate was unacceptably high (22%) with radiotherapy alone using 50 Gy in the GROINSS-V-II study [21, 22]. Another important element is lymphovascular invasion (LVSI), as demonstrated by Serre et al. [23], LVSI is an independent negative risk factor for recurrence-free survival even in women with single intracapsular lymph node metastasis.

In high-risk lymph-node-positive women, radiotherapy improved local control, relapse-free survival, and overall survival (OS) [24, 25]. AGO-CARE-1 showed that the local recurrence rate significantly reduced from 25.5% in lymph-node-positive patients without adjuvant RT to 15.8% in lymph-node-positive patients with adjuvant RT to the vulva and groins/pelvis (HR 1.79; *p* = 0.019), independent of the resection margin status [26]. Additionally, there was greater impact with adjuvant radiotherapy for HPV-related tumors than for HPV-independent tumors with a median disease-free survival of 20.7 months versus 17.8 months, respectively [26]. Although the results of these studies are in favor of post-operative radiotherapy, these data have been mainly derived from retrospective studies in which the treatment modalities were not the same. Due to the rarity of vulvar tumor, the complexity of treatments, and the possible iatrogenic damages, the optimal adjuvant therapy for women with vulvar cancer remains controversial, as it has been poorly described and there is a paucity of evidence in the literature.

## Methods

### Study Design

This is a systematic review on the efficacy of adjuvant radiotherapy on patients with histologically confirmed squamous vulvar cancer and groin metastasis. The main objective of this review is to assess the effectiveness and safety of adjuvant radiotherapy to locoregional nodal areas for women diagnosed with node-positive vulvar cancer who had undergone surgical treatment (which included removal of groin lymph node/s). The method of research follows the 2020 PRISMA statement [27].

### Inclusion Criteria

The study aimed to ask the following PICOS items. Population: The study included patients diagnosed for squamous vulvar cancer who had undergone primary vulvar surgery and groin lymph node dissection with histologically confirmed cancer involving the lymph nodes. Intervention: adjuvant radiotherapy following surgical intervention on the vulva and groins. Comparators: the control group is represented by patients treated surgically for carcinoma of the vulva with inguinal lymph node metastases who did not undergo adjuvant radiotherapy treatment. Outcome: primary outcomes are OS at 5 years (survival from randomization to death from any cause), cancer-related death rate, disease-free survival at 12 months, site of recurrence or relapse, and acute toxicities from adjuvant treatment (e.g., wound infection). Secondary outcomes are late toxicities from adjuvant treatment and quality of life (QoL) following treatment. Study design: we included only randomized controlled studies (RCTs) on the subject. If the results from research are poor, we will also add a narrative review describing the results of well-conducted retrospective studies. The review includes only articles in English.

### Search Strategy

We conducted a comprehensive and systematic literature search of MEDLINE (1946 to present), Embase.com (1974 to present), Cochrane Central Register of Controlled Trials (CENTRAL), Google Scholar (2004 to present), ClinicalTrials.gov^1^, and the National Cancer Institute. A combination of words was used as follows: “Vulvar cancer” OR “Squamous vulvar cancer” AND “radiotherapy” OR “adjuvant radiotherapy.” The systematic review protocol was registered in the International prospective register of systematic reviews—PROSPERO (CRD42023495140).

**FIGURE 1:**
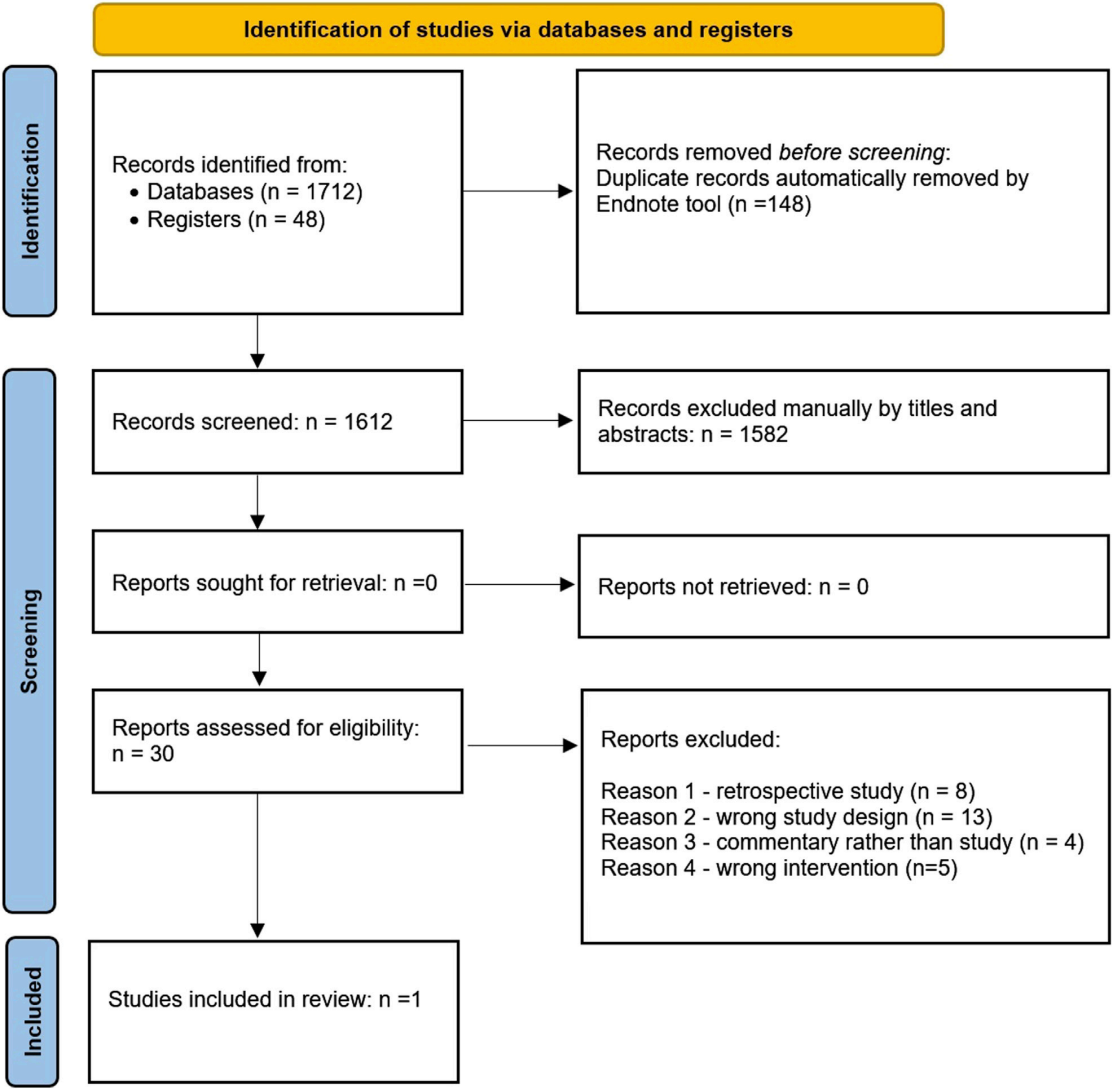
PRISMA flow diagram.

We downloaded all titles and abstracts retrieved by electronic searching into the reference management database (EndNote). Three review authors (HS, LI, and EG) independently removed duplicates and examined the remaining references. Independently, two review authors (FF and AS) assessed the eligibility of the review articles. Disagreements were resolved by discussion between the two review authors or involved a third author (RO). We selected publications on the effectiveness of adjuvant radiotherapy treatment for squamous cell carcinoma of the vulva and excluded studies that clearly did not meet the inclusion criteria. We recorded the selection process in sufficient detail to complete a PRISMA flow diagram (Figure 1).

### Data Extraction and Analysis

For included studies, we extracted data as recommended in Chapter 7 of the Cochrane Handbook for Systematic Reviews of Interventions [28]. This included data on trial information (author, year of publication, country, setting, inclusion and exclusion criteria, study design, methodology, study population, and total number enrolled), patient characteristics (age, comorbidities, primary treatment, grade and stage of vulvar cancer, excision margins, and depth of invasion), details of adjuvant treatment received (dose, fraction size, field, type of radiotherapy, and timing), type of assessment measures used (generic or disease specific, or a combination), reporting of complications/toxicity from adjuvant therapy, duration of follow-up, response to treatment, and identification of disease recurrence. We extracted results as follows: for time-to-event data, we extracted the hazard ratio (HR) and its confidence interval from the trial report; for dichotomous outcomes, if it was not possible to use a HR, we extracted the number of participants in each treatment arm who had experienced the outcome of interest and the number of participants assessed at end point, in order to estimate a risk ratio (RR). We noted the time points at which outcomes were collected and reported. Two review authors (HS and LI) independently abstracted the data. Differences between review authors were resolved by discussion or by an appeal to a third review author (FF).

### Assessment Risk of Bias

We assessed the risk of bias in the included RCT using the Cochrane “risk of bias” tool and the criteria specified in Chapter 8 of the Cochrane Handbook for Systematic Reviews of Interventions [29]. This includes assessment of bias arising from the randomization process, bias due to deviations from intended interventions, bias due to missing outcome data, bias in measurement of the outcome, and bias in selection of the reported result [30]. Two review authors (FF and EG) applied the risk-of-bias tool independently and differences were resolved by discussion or by appeal to a fourth review author (HS). The only study included in the review was judged at low risk of bias.

## Results

We identified 1,760 records from database and register searches. Two authors (FF and AS) excluded non-relevant and duplicated records from title and abstract screening and retrieved 30 records for full-text review. From this, we identified one eligible randomized control trial that warranted full evaluation [31]. This study met the defined inclusion criteria and addressed most of our desired outcomes, although there was no information pertaining to QoL.

### Study Design, Setting, and Participants

The included study was part of GOG protocol #37, conducted at Cleveland Hospital, Ohio, United States. The study included 114 women with primary invasive squamous cell carcinoma of the vulva, whose primary lesions and groin nodes were amenable to radical vulvectomy and bilateral inguinal lymphadenectomy, regardless of the International Federation of Gynecology and Oncology (FIGO) staging (FIGO 1969). All women underwent radical vulvectomy and bilateral groin node resection; surgical excision of bilateral groin nodes included both superficial and deep nodes relative to the inguinofemoral fascia, including nodal skeletonization of the femoral artery and vein. Nodal tissue medial to the femoral artery and vein was designated the Cloquet node and surgically excised. Women who were determined to have unilateral or bilateral groin metastases at the time of surgery were then intraoperatively randomized (by central telephone allocation) to either ipsilateral pelvic node resection or pelvic and groin radiation. The exclusion criteria were recurrent disease, prior malignancies, positive groin nodes not resected at surgery, or women deemed unsuitable for radiation treatment. The intention of the study was to detect a 20% or greater increase in survival after radiation, as compared to the baseline survival estimate of 40%. To achieve this sensitivity with a power of 0.80 and a significance level of 0.05, it was determined that 76 women would be required in each arm of the study. However, because of an observed statistical superiority of the addition of radiotherapy, the study was closed approximately 2 years short of the projected accrual goals. Fifty-five women were randomized to pelvic node resection, which followed a standardized extraperitoneal approach, enabling excision of the external iliac, internal iliac, obturator, and common iliac nodes. Fifty-nine women were randomized to pelvic radiation, the fields of which encompassed both groins, and obturator, external, and internal iliac nodal areas. No central vulvar radiation was administered. Radiotherapy began within 6 weeks of vulvectomy. Women received 45–50 Gy to the pelvic midplane, halfway between the superior border of the obturator foramina and the L5–S1 interspace, at a daily fraction of 1.8–2 Gy. Dose calculations were made at the center of the inguinal and femoral node areas at a depth of 2–3 cm from the anterior body surface. Anterior–posterior and posterior–anterior fields were treated each day for 4.5–6 weeks. The number of positive groin nodes, histopathological grade, depth of invasion, maximum tumor dimension, and tumor lymph vascular invasion were similar among the radiation and pelvic node resection arms, and there was no significant difference in the distribution of clinical groin node status between the two arms.

### Primary and Secondary Outcomes

To establish the main outcomes, cancer-related death was calculated from the date of study entry to the date of physical or radiographic evidence of cancer-specific death or date last seen. Recurrence-free survival was defined as the period from the date of study entry to the date of physical or radiographic evidence of recurrent vulvar cancer. Women were followed up for at least 6 years; data on OS were reported at 2 and 6 years of follow-up. At 2 years, the OS was 68% in the radiation group compared to 54% in the pelvic node resection arm. The study was closed early based on the results. Updated analysis revealed a 6-year OS of 51% versus 41% in favor of radiotherapy compared to pelvic node resection; the study did not report if this finding was statistically significant. This reduction in OS benefit from 2 years to 6 years was largely because of a high number of non-cancer deaths in the radiotherapy arm after 2 years (14 vs. 2). However, the long-term follow-up supported that patients with N2/3 disease benefited significantly from radiation treatment, and indeed, when women with over 20% positive groin nodes were considered separately, the OS at 6 years in those treated with radiation was 36% compared with 13% in those treated with pelvic node resection. At 6 years of follow-up, the cumulative incidence of cancer-related deaths was 29% in the radiation arm compared with 51% for pelvic node resection (HR 0.49; 95% CI 0.28–0.87; low- certainty evidence). RFS at 6 years was estimated to be 48% after pelvic node resection compared with 59% after radiation. Interestingly, all 27 vulvar cancer recurrences recorded after pelvic node resection occurred within 24 months, while 4 of 21 recurrences after radiation occurred after 24 months. The dominant pattern of recurrence in the radiant arm was distant metastasis (7/57 patients), while the main site of recurrence in the control arm was the groins (13/54 patients). Acute toxicities were assessed as those occurring postoperatively or within 60 days of protocol treatment. There was no significant difference in the proportion of women experiencing wound infection (61 vs 56%), urinary tract infection (11 vs 11%), pulmonary embolism (0 vs 2%), stroke (2 vs 0%), sepsis (11 vs 5%), or acute lymphedema (30 vs 25%) after pelvic node dissection compared to radiation. The late cutaneous toxicity rate was 19% after radiotherapy and 15% after pelvic node resection (*p* = 0.62). The median time to late persistent grade 2 or higher cutaneous toxicity was 60 days (range 9–499 days) after radiation and 80 days (range 29–202 days) after pelvic node resection. The chronic lymphedema rate was 16% after radiation and 22% after pelvic node resection (*p* = 0.47). The median time to late persistent grade 2 or higher lymphedema was 202 days (range 15–715 days) after radiation and 192 days (range 86–799 days) after pelvic node resection. The study did not investigate the QoL of the included patients. The main results are presented in the summary of findings tabulated in Table 1. The only study included in the review was judged at low risk of bias; the traffic light graph, weighted bars, and the authors’ comments for each domain can be found in the Supplementary Material [32]. We provided the source and rationale for each assumed risk cited in the table and used the GRADE approach to rank the quality of evidence using the GRADE Profiler Guideline Development Tool software (GRADEpro GTD) [33].

**TABLE 1:** Summary of findings.

Outcome	Anticipated absolute effects* (95% CI)		Relative effect (95% CI)	No. of participants (studies)	Certainty of the evidence (GRADE)	Comments
	Risk with ipsilateral pelvic node resection (with no radiotherapy)	Risk with adjuvant radiotherapy				
Overall survival (OS) assessed with: months; follow-up: 72 months	407 per 1,000	273 per 1,000 (145–493)	HR 0.61 (0.30–1.30)	111 (1 RCT)	⊕○○○ Very low^a^ ^,^ ^b^ ^,^ ^c^	The evidence suggests adjuvant radiotherapy may improve OS in patients with groin metastasis
Recurrence-free survival (RFS) assessed with: number of events; follow-up: 72 months	481 per 1,000	226 per 1,000 (106–439)	HR 0.39 (0.17–0.88)	111 (1 RCT)	⊕⊕○○ Low^a^ ^,^ ^b^	The evidence suggests adjuvant radiotherapy results in a slight increase in RFS
Cancer-related deaths (CRD) assessed with: number of events; follow-up: 72 months	519 per 1,000	301 per 1,000 (185–471)	HR 0.49 (0.28–0.87)	111 (1 RCT)	⊕⊕○○ Low^a^ ^,^ ^c^	The evidence suggests adjuvant radiotherapy results in a reduction in CRD
Acute toxicities from adjuvant treatment (within 60 days of treatment): Wound infection	611 per 1,000	561 per 1,000 (409–764)	RR 0.918 (0.670–1.250)	111 (1 RCT)	⊕○○○ Very low^a^ ^,^ ^b^ ^,^ ^c^	The evidence is very uncertain regarding the effect of adjuvant radiotherapy on wound infection
Late toxicities from adjuvant treatment: cutaneous toxicity (Grade 2 or more); assessed with: number of events; follow-up: 6 years	148 per 1,000	197 per 1,000 (84–443)	RR 1.33 (0.57–2.99)	111 (1 RCT)	⊕○○○ Very low^a^ ^,^ ^b^ ^,^ ^c^	Adjuvant radiotherapy may have little to no effect on cutaneous toxicity, but the evidence is very uncertain
Late toxicities from adjuvant treatment: lymphedema (grade 2 or more); assessed with: number of events; follow-up: 6 years	222 per 1,000	158 per 1,000 (71–344)	RR 0.71 (0.32–1.55)	111 (1 RCT)	⊕○○○ Very low^a^ ^,^ ^b^ ^,^ ^c^	The evidence is very uncertain regarding the effect of adjuvant radiotherapy on development of post-treatment lymphedema
Site of recurrence: groin; assessed with: number of events; follow-up: 72 months	241 per 1,000	52 per 1,000 (16–173)	RR 0.218 (0.065–0.720)	111 (1 RCT)	⊕⊕○○ Low^a^ ^,^ ^c^	Adjuvant radiotherapy may result in a large reduction in cancer recurrence on the groin

*The risk in the intervention group (and its 95% confidence interval) is based on the assumed risk in the comparison group and the relative effect of the intervention (and its 95% CI).

CI, confidence interval; HR, hazard ratio; RR, risk ratio.

GRADE working group grades of evidence

High certainty: we are very confident that the true effect lies close to that of the estimate of the effect.

Moderate certainty: we are moderately confident in the effect estimate: the true effect is likely to be close to the estimate of the effect, but there is a possibility that it is substantially different.

Low certainty: our confidence in the effect estimate is limited: the true effect may be substantially different from the estimate of the effect.

Very low certainty: we have very little confidence in the effect estimate: the true effect is likely to be substantially different from the estimate of effect.

Explanations

^a^ Downgraded due to the lack of ability to measure consistency as only one study was identified.

^b^ The confidence interval is wide, study population includes a small sample, and the number of events is low.

^c^ The trial was closed before accrual.

### Narrative Review

In this section, we report the main results of the retrospective studies we examined during the selection process. The common point among the following papers is that they aimed to investigate the possible role of adjuvant radiotherapy treatment after surgery for patients with squamous cell carcinoma of the vulva and positive lymph nodes. Beyond this objective, the populations under investigation and the treatments received, however, are characterized by wide heterogeneity.

In 2012, Woelber et al. [5] conducted a single-center retrospective analysis of approximately 157 consecutive patients with primary squamous cell cancer of the vulva; all patients underwent the same primary surgery consisting of the triple-incision technique surgery on the vulva and groins with the result of complete tumor resection in all cases. Forty-nine patients had lymph-node metastasis, of which 21 (42.9%) had 1, 13 (26.5%) had 2, and 15 (30.6%) had more than 2 affected nodes. Adjuvant radiotherapy was administered to 32% of patients of all the samples: radiation fields included the vulva in 12 patients and the groins/pelvis in 11 patients; in 26 patients, both fields were irradiated. In 7 of the 37 patients with irradiation to the groins/pelvis, only the inguinal field was irradiated. Of the patients with only 1 positive node, 14 received adjuvant radiotherapy, of which 7 were because of a nodal metastasis of greater than 10 mm or with an extracapsular spread. In the remaining patients, adjuvant therapy was indicated because they were judged at high risk of recurrence by the interdisciplinary tumor board. The inguinal/pelvic radiation dose was prescribed uniformly; cumulative doses ranged between 50.4 and 59.4 Gy. The multivariate analyses showed that all patients with positive nodes were at significantly higher risk for disease recurrence irrespective of the number of nodes affected (*p* ≤ 0.029). The effect of positive nodes differed, depending on the adjuvant treatment: in patients without adjuvant radiotherapy to the groins/pelvis, the number of tumor-involved nodes was highly relevant for prognosis (HR 1.752; *p* ≤ 0.001), whereas in patients with adjuvant radiotherapy, the HR of an additional positive node was decreased by 45% (*p* = 0.001), losing its statistical relevance (HR 0.972; *p* = 0.828). The main conclusions of the author was that in patients receiving adjuvant radiotherapy, the negative effect of additional lymph node metastases is reduced and that adjuvant treatment might therefore be beneficial even in patients with only one positive node [5].

In 2018, Rydzewski et al. [34] published a retrospective article involving 2,779 women with squamous vulvar carcinoma with pathologically confirmed positive lymph nodes after surgical treatment of the vulva and groins. After primary surgery, 1,061 patients (38%) received no adjuvant treatment, 974 (35%) received adjuvant external beam radiation therapy (EBRT), and 744 (27%) received adjuvant chemoradiotherapy (CRT). Patients who received no adjuvant treatment more often had one (58.4%) positive node, while patients who received adjuvant CRT more often had two or more (57.8%) positive nodes. Significant predictors of receipt of EBRT were the presence of two or more positive nodes and living closer to the hospital, while significant predictors of receipt of CRT were having two or more positive nodes, being younger in age, having fewer comorbidities, and being diagnosed in more recent years. The results showed that 5-year OS was highest among patients with one positive node who received CRT (68.1%), compared to 55.9% for those who received adjuvant EBRT and 46.1% for those with no adjuvant treatment. Survival was likewise highest among patients with two or more positive nodes who received CRT (49.1%), compared to 29.4% for those who received adjuvant EBRT and 21.2% for those with no adjuvant treatment. The univariate analysis found significantly decreased mortality for patients with one positive node who received EBRT (HR 0.72; *p* = 0.001) versus no adjuvant treatment, patients with two or more positive nodes receiving EBRT (HR 0.64; *p* < 0.001) versus no adjuvant treatment, patients with one positive node receiving CRT (HR 0.68; *p* = 0.004) versus adjuvant EBRT, and patients with two or more positive nodes receiving CRT (HR 0.61; *p* < 0.001) versus adjuvant EBRT. At multivariate analysis, the significant reduction in HR for adjuvant EBRT compared to no adjuvant treatment persisted (HR 0.81; *p* = 0.027), and in HR for adjuvant EBRT compared to no adjuvant treatment in patients with two or more positive nodes (HR 0.59; *p* < 0.001). For patients with one positive node receiving CRT, the statistical significance of survival advantage compared to EBRT was lost in the multivariate analysis (HR 0.93; *p* = 0.605), while the benefit persists in women with two or more positive nodes who received CRT instead of EBRT (HR = 0.79; *p* = 0.022) [34].

The AGO-CaRE-1 study analyzed the effect of administering adjuvant therapy to patients with lymph-node-positive primary or recurrent squamous vulvar cancer after surgical treatment. Surgery included wide local excisions, partial vulvectomy, complete vulvectomy, and pelvic exenteration. The sample included 1,249 patients from 29 centers, 447 of whom had positive lymph nodes (N+): 54.6% of N+ patients received adjuvant therapy and the majority (84.4%) was treated with RT, while 13.5% received concomitant CRT. The most frequently applied cytostatic agent was cisplatin (in 72.7% as single agent and in 6.1% as combination therapy). RT was applied heterogeneously, particularly in terms of treatment volume, and included the inguinal nodes in 183 of the 239 patients (40.9% of all 447 node-positive patients) with adjuvant CRT and both the inguinal and pelvic nodes in 117 of 239 cases, the latter being a subset of the 183 patients. Sixty-six of the 239 patients received adjuvant therapy to the inguinal nodes without a pelvic field. Target-specific doses were not specified. The median total dose applied in all N+ patients with adjuvant radiotherapy regardless of the fields irradiated was 50.4 Gy. The 3-year PFS rate in N+ patients receiving adjuvant therapy was statistically significantly better than that in N+ patients without adjuvant treatment (39.6% vs. 25.9%; HR = 0.67; 95% CI = 0.51–0.88, *p* = 0.004), whereas the difference in the 3-year OS rate was statistically not significant (57.7% vs. 51.4%; HR = 0.79; *p* = 0.17). Looking at adjuvant therapy by univariate subgroup analysis about the number of affected nodes, the PFS rate was statistically significantly lower for patients with adjuvant RT in case of two or more affected nodes. The HR was 0.44 in patients with two positive nodes (*p* = 0.004), 0.37 in patients with three positive nodes (*p* = 0.004), and 0.45 in patients with more than three positive nodes. In multivariable analysis of the node-positive patients with adjuvant radiotherapy directed to the groins+/pelvis+/vulva and those without adjuvant radiotherapy adjusted for age, ECOG (Eastern Cooperative Oncology Group) scale, stage, grade, invasion depth, and number of positive nodes, the effect of adjuvant therapy on PFS and OS remained consistent (PFS: HR = 0.58; *p* < 0.001) [35].

The multicenter, retrospective study OLDLADY-1.2 aimed at assessing the efficacy and safety of adjuvant radiotherapy in vulvar cancer patients treated in nine Italian radiation oncology institutions, covering a 20-year time interval (February 2000–November 2019). Seventy-three (40.4%) patients underwent wide local excision or deep partial vulvectomy and 108 (59.6%) underwent total deep vulvectomy according to the glossary of terminology proposed by Micheletti et al. [36]. Unilateral and bilateral IFL was performed for 17 (9.3%) and 141 (77.9%) patients, respectively, while 5 (2.7%) patients received sentinel node dissection (SND). Adjuvant treatment was administered according to the presence of the following risk factors: inguinal positive lymph nodes, tumor diameter larger than 4 cm, and positive or close margins and depth of invasion deeper than 5 mm. Sixty-one (33.7%) patients received adjuvant CRT and 120 (66.3%) received RT alone. All patients with positive lymph nodes received adjuvant treatment, so there was no control group with which the OS or DFS data could be compared. The study reported a wide heterogeneity in radiation doses and volumes, according to the status of margin, and the presence of pelvic or inguinal nodal involvement. The primary study end point was the 2-year local control, and the secondary end points were the 2-year metastasis-free survival, the 2-year OS, and the rate and severity of acute and late toxicities. With a median follow-up of 27 months (range 1–179 months), the 2-year actuarial local control rate, metastasis-free survival, and OS were 68.7%, 84.5%, and 67.5%, respectively. In the adjuvant therapy group, there were 45 (37.5%) tumor bed recurrences and 23 (19.1%) lymph node failures versus 16 (26.2%) tumor bed and 10 (16.3%) nodal relapses in patients treated by CRT [37].

Ni et al. [38] conducted a retrospective study that included 2,396 patients aged 65 years and older with pathologic node-positive vulvar cancer, of whom 1,517 (63.3%) received adjuvant RT. The group of patients who received adjuvant RT differed from the group that received surgery alone with respect to age, race, income quartile, residential setting, distance from the facility, year of diagnosis, and nodal status. In particular, patients who underwent surgery alone were older than patients who received surgery with RT (median 78.9 years vs. 75.9 years, *p* < 0.001), were more likely to be White than non-White race (*p* = 0.005), to be from zip codes associated with lower income quartiles (20.4% vs. 17.5% in the lowest income quartile, *p* = 0.001), to live in rural and urban settings compared to metropolitan areas (*p* = 0.01), and to live farther distances from the treating facility (*p* < 0.001). Patients who were diagnosed in more recent years (*p* = 0.001) and those with pathologic N2 disease compared to N1 disease (*p* < 0.001) were more likely to receive adjuvant radiotherapy. The OS for the entire cohort was 32% at 5 years; on univariate analysis, surgery with adjuvant RT was associated with a significant improvement in OS when compared to surgery alone (5-year OS 35% vs. 26%; *p* < 0.0001). After controlling for demographic, clinical, and treatment differences on multivariate analysis, adjuvant RT continued to be significantly associated with improved OS (OR 0.78; CI 0.70–0.87; *p* < 0.001). In the propensity score-matched cohort, the OS was 29% at 5 years; the 5-year OS was 33% among patients who received adjuvant RT compared to 26% among patients who did not receive adjuvant RT (*p* < 0.0001); and on multivariate analysis, adjuvant RT continued to be associated with significantly improved OS (OR 0.77; CI 0.69–0.87; *p* < 0.001) [39].

The study by Li et al. retrospectively analyzed 3,571 patients who underwent surgery and were diagnosed with vulvar squamous cell carcinoma between 2010 and 2015, extrapolating data from the Surveillance, Epidemiology, and End Results Program (SEER) database [37]. A propensity score matching (PSM) approach was used to balance the differences in clinicopathological characteristics between the group that received RT as adjuvant treatment and the group that did not. The multivariate analysis showed that postoperative RT had no significant effect on OS (HR 1.138; 95% CI 0.940–1.377; *p* = 0.186) and DSS (HR 1.006; *p* = 0.959) of patients, while age, race, AJCC staging, N staging, and tumor size were independent factors affecting patients’ OS and DSS. The subgroup analysis showed a significant benefit of postoperative radiotherapy in improving the OS in patients with AJCC grade III and N1 (*p* = 0.048 and *p* = 0.004, respectively) and for patients with large tumor size (>3.5 cm) (*p* = 0.021) [36].

Bruce et al. [39] conducted a retrospective cohort study on 201 women with squamous vulvar carcinoma treated with primary surgery only versus surgery with adjuvant radiation or primary radiation. Fifty-one (25.4%) women underwent primary radiation, whereas 150 (74.6%) women underwent primary surgery. The median follow-up time was 3.3 years. The median external pelvic radiation dose was 45 Gy; 63% (32/51) of the primary radiation patients also received interstitial vulvar brachytherapy at a median dose of 18 Gy. Ten (10/51, 19.6%) women underwent surgical resection of residual disease after primary radiotherapy. Among the primary surgery group, 114 (76.0%) women were treated with surgery alone. Thirty-six (24.0%) women received adjuvant RT with or without chemotherapy. The median adjuvant pelvic radiation dose was 50.4 Gy; indication for adjuvant radiation was at the discretion of the treating surgeon and was not reliably documented. The target field for adjuvant radiation most often included both the vulva and nodes, but select patients received nodal doses only if their primary tumor was completely resected with negative margins. Treatment groups had no significant differences in age, Charlson Comorbidity Index, or smoking status. Surgical patients were at a lower stage; 93.0% of surgery-only patients were at stage I, whereas 83.0% of primary radiotherapy patients were at stage II–IV (*p* < 0.001). The statistical analysis showed that patients in the primary surgery-only group had significantly better crude OS and PFS than patients in the primary surgery + adjuvant RT and primary RT groups (3-year OS 82.6% for PS alone, 48.3% for PS + RT, and 53.9% for PRT; *p* < 0.001). The authors have commented these results underling that favorable survival in the primary surgery-only group was likely attributable to lower stage cancers, as survival advantage was not observed in multivariable analysis controlling for stage. The OS and PFS in the primary surgery + RT and primary RT were similar in bivariate analyses and multivariable analysis controlling for stage. The authors concluded that survival is most impacted by stage rather than primary treatment modality [39].

Finally, we report the results of Van Der Velden et al.’s [40] study on 96 patients with single clinically occult intracapsular lymph node metastasis treated with no adjuvant radiotherapy after surgical procedure. All patients underwent radical local excision of the primary tumor and either unilateral or bilateral IFL. The median follow-up was 64 months (range 7–248 months). Recurrence occurred in 40 (41.7%) of 96 patients: in 27 cases, recurrence was local; in one case, it occurred at the groin; in three cases, it was found in the pelvis; in three cases, it was a distant metastasis; in two cases, the recurrence was both local and at the groin; and in four cases, it was both local and distant metastasis. The study demonstrated a very low risk of an isolated groin recurrence in patients with squamous cell cancer of the vulva and a single clinically occult intracapsular positive lymph node after IFL without adjuvant radiotherapy. Only one patient showed an isolated groin recurrence (on the contralateral side). The authors found that neither the size of the metastasis in the lymph node (<5 vs. ≥5 mm) nor the lymph node ratio had any impact on the groin recurrence rate and/or survival in this group of patients [40].

## Discussion

In the study included in this review, the main objective was to demonstrate the benefit of receiving radiotherapy instead of surgery alone, and the study design with a 20% or greater survival benefit after radiotherapy was desired. To achieve this, 76 women per treatment arm were required. However, because of a perceived superiority of radiotherapy, the study was closed early (after 2 years) prior to the accrual of the optimal sample size. At 2 years of follow-up, the OS benefit of radiotherapy was 14%. At 6 years, this benefit had reduced to 10%, but when assessed for cancer-related death at 6 years, the cumulative incidence was 29% for radiotherapy compared with 51% for pelvic node resection. Patients’ age and comorbidities are the main explanations to the reduction of benefit on OS from 2 years of follow-up and 6 years of follow-up. This can be attributed to the fact that most patients died of non-cancer-related causes as found by Ni et al. [38] in their retrospective work: the benefit of adjuvant radiotherapy is lost in patients older than 85 years. These survival data and outcomes must be viewed cautiously, as the trial may have been underpowered and, because of this, may have contributed to gaining only very low certainty of evidence. Another limitation of this work that must be considered is that it used FIGO 1969 staging without distinguishing between micro- and macrometastases, identifying patients with N2/3 or over two metastatic lymph nodes as the category that benefits most from adjuvant treatment. Additionally, the results of this work predate the sentinel lymph node era. Despite this, the key message of the study is that patients’ prognosis remains linked to the number and type of lymph node metastases and the treatment received, as also shown in the retrospective studies above [34, 35]. This concept is consistent with the recent findings on this neoplasm: the GROINS-V-II study established inguinofemoral RT without surgical groin dissection as a safe alternative to inguinofemoral lymph node dissection for patients with sentinel lymph node micrometastases but could not make similar conclusions for patients with SN macrometastasis. In patients with macrometastasis treated by radiotherapy alone without inguinofemoral lymph node dissection, the isolated groin recurrence rate at 2 years was 22% versus 6.9%, suggesting that patients with sentinel lymph node macromestasis should receive both inguinal lymph node dissection and adjuvant radiotherapy treatment [22].

The result on local recurrence found in the RCT is in agreement with the sub-analysis of the AGO-CARE-1 study on 360 node-positive patients with vulvar squamous cancer, FIGO stage ≥IB, treated by primary surgery and observation alone versus radiotherapy on the vulva and groins/pelvis versus radiotherapy on the groins/pelvis. The retrospective analysis showed that recurrence at the vulva occurred in 25.5% of patients without adjuvant RT, in 22.8% of patients with adjuvant RT to the groins/pelvis, and in 15.8% of patients with adjuvant RT to the vulva and groins/pelvis; interestingly, 50% disease-free survival time (50% DFST) showed a stronger impact of adjuvant RT to the vulva in HPV+ compared to HPV− patients (50% DFST 20.7 months vs. 17.8 months) [26]. Regarding short- and long-term toxicities, these occurred in similar proportions in both the radiotherapy and surgery-only groups: the acute lymphedema rate was 30% after pelvic node resection and 25% after radiation, while chronic lymphedema occurred in 16% of patients after radiation and in 22% after pelvic node resection. Similar results in the two groups suggest that the cause of lymphedema is the removal of the inguinal lymph nodes; in the study, however, it is not shown whether the saphenous vein was preserved during lymphadenectomy as suggested in other studies, to reduce this post-operative complication [37]. Acute and chronic lymphedema have a strong impact on QoL in cancer patients [36], so in order to minimize this collateral effect, we are awaiting the results of the ongoing GROINSS-V-III (NRG-GY024) study, in which patients with stage I, unifocal, invasive (>1 mm depth of invasion) squamous cell carcinoma of the vulva with tumor size <4 cm and macrometastatic disease in the SLN will have adjuvant radiotherapy to the groin(s) at an increased dose of 56 Gy combined with concurrent cisplatin chemotherapy. The hypothesis is that the addition of cisplatin chemotherapy and the increased radiation dose to the groins will prevent groin recurrences and avoid the need for a full IFL in patients with macrometastasis in the SLN [41]. The narrative description shows how we can derive information from studies that are difficult to compare with each other due to different numbers, different surgical approaches to the initial neoplasm, different dosages and volumes used for radiotherapy treatments, and the possible addition of a radiosensitizing chemotherapeutic agent [34]. To make the picture even more complicated, it remains a fact that there is no unequivocal indication for prescribing adjuvant treatment [38], which makes the population under review even more heterogeneous. This could explain why the indications for adjuvant treatment that we find in the guidelines of the main scientific societies report a low level of evidence. Actually, ESGO and NCCN guidelines recommend adjuvant radiotherapy on the groin in case of micrometastasis in SLND, macrometastasis in SLND after IFL, more than one node metastasis after IFL is performed at primary surgery, or in case of extranodal extension [8, 9]. In addition to the presence of positive lymph nodes, factors such as large primary tumors, deep invasion of the stroma, lymphovascular invasion, and narrow margins of surgical resection are linked to a greater risk of the disease coming back, therefore the guidelines suggest that these elements should be considered in order to consider the indicated adjuvant radiotherapy treatment. The Gynecologic Oncology Group (GOG) suggests that adjuvant radiation should be the norm for treating vulvar squamous cell carcinoma in patients who have two or more lymph nodes affected with extracapsular extension or when inguinofemoral dissection is not an option. The advantage of adding radiotherapy has been confirmed in cases with at least two positive inguinofemoral lymph nodes. However, the effectiveness of radiation therapy in cases with a single positive inguinofemoral lymph node still remains uncertain [42, 43].

### Quality of Evidence

We judged the information in the trial to be of very low certainty of evidence. As seen in the summary of findings (Table 1), much of the examined data also displayed wide confidence intervals, and it is for this reason that all outcomes examined were downgraded by at least one level to reflect a degree of imprecision. There was a further downgrade due to potential inconsistency due to sparsity of data. However, there was no other significant risk of bias or indirectness to clearly justify further downgrading of the presented evidence.

## Conclusion

This randomized controlled trial (RCT) was aimed to identify a 20% improvement in survival rates with the use of radiotherapy. However, the enrollment was prematurely halted following an early analysis showing a statistical advantage for radiotherapy. Although this advantage in survival diminished at a 6-year follow-up, it still seemed to remain after adjusting for deaths not related to cancer. The study also provided guidelines for radiotherapy in women with more than 20% positive ipsilateral groin nodes (ratio of positive nodes detected to the number of groin nodes removed). According to the trial data, a node positivity rate of over 20% appeared to be linked with contralateral groin and pelvic nodal metastases, recurrence, cancer-related deaths, and OS. The research suggested that women with a node positivity rate of over 20% could be considered for adjuvant groin and low pelvic radiotherapy, as it appears to lessen the chance of recurrence.

The review is based on very low quality evidence; hence all findings should be approached with caution. Our analysis seems to indicate that nearly all primary outcomes, including OS, recurrence-free survival, cancer-related death, recurrence (groin), and acute toxicities, were improved in women who received adjuvant radiotherapy. However, women who underwent radiotherapy seemed to experience higher late (cutaneous—grade 2 or higher) toxicity than those who only had surgery. These results imply that adjuvant radiotherapy is beneficial, but it is important to note that the evidence level was low to very low for all outcome parameters, causing cautious interpretation.

## Data Availability

The original contributions presented in the study are included in the article/Supplementary Material; further inquiries can be directed to the corresponding author.

## Footnotes

1
www.ClinicalTrials.gov
