# Supplementary material for: Adjuvant Radiotherapy for Groin Node Metastases Following Surgery for Vulvar Cancer: A Systematic Review
Source: Oncol Rev. 2024 May 7;18:1389035. doi: 10.3389/or.2024.1389035 (PMC11107452; doi:10.3389/or.2024.1389035)
Supplement: Supplementary file 1 [file DataSheet1.DOCX]

**Supplementary material**


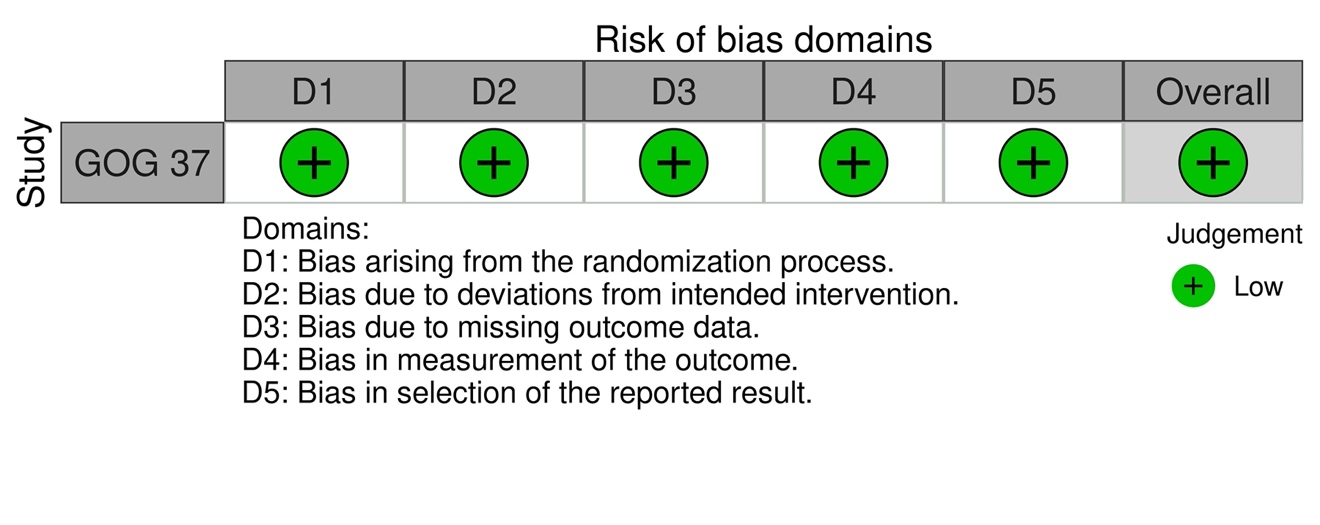


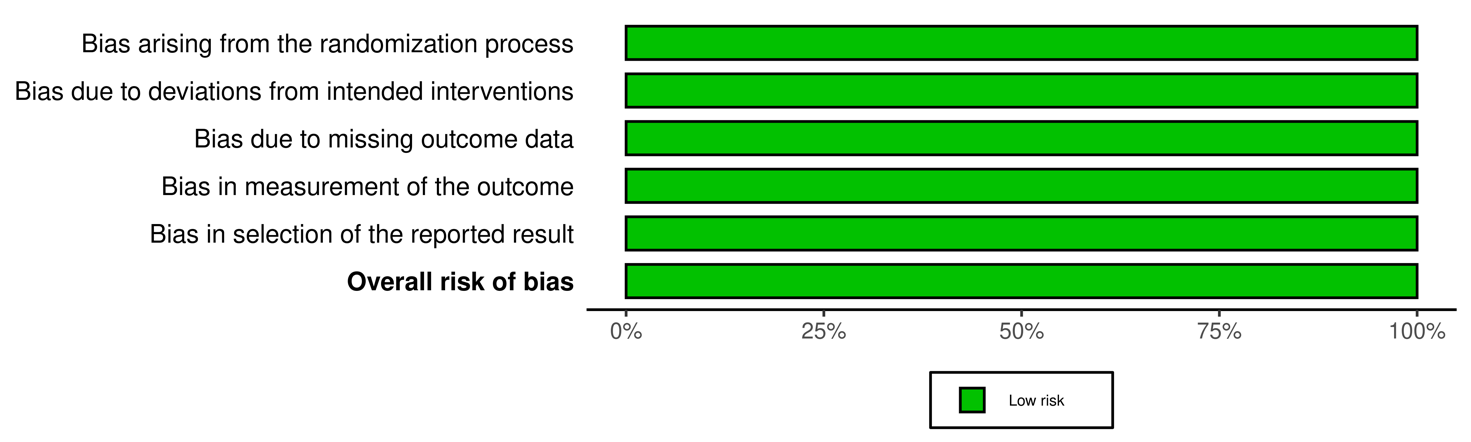
Comments:

**Domain 1 (bias arising from the randomization process):** All the women enrolled in the study were randomized at the time of surgery by central telephone allocation to either ipsilateral pelvic node resection or pelvic irradiation.

**Domain 2 (bias due to deviations from intended interventions):** Partecipants were aware because intervention group was represented by adiuvant radiotherapy vs surgery alone. Due to the nature of the intervention, blinding to the patients and clinicians were not possible. Randomization was made by central telephone allocation at the time of surgery.

**Domain 3 (Bias due to missing outcome data):** The women were excluded from the analysis owing to incomplete or missing data. Data on long-term adverse effects to the treatment arm was only available in a total number of 57 women at two years due to participant death. The risk of bias from this is felt to be low.

**Domain 4 (Bias in measurement of the outcome):** All patients' outcome were recorded in detail. Primary end-points of the study were overall survival at six years, recurrence free-survival and acute al late toxicity related to the two treatment arms. There was no information available on quality of life. The risk of bias is felt to be low.

**Domain 5 (bias in selection of the reported results):** The numbers of positive groin nodes, histopathological grade, depth of invasion, maximum tumour dimension and tumour lymphovascular invasion were similar across the two groups. Whilst baseline characteristics were not detailed in the paper adjustment was made for age, treatment, adverse tumour characteristics, BMI, pre-radiation haematocrit and medical comorbidities did not significantly affect survival (P > 0.05 for all).
